# A Rare Lymphoproliferative Disease: Castleman Disease

**DOI:** 10.4274/tjh.galenos.2021.2021.0440

**Published:** 2021-12-07

**Authors:** Eren Gündüz, Nihal Özdemir, Şule Mine Bakanay, Sema Karakuş

**Affiliations:** 1 Eskişehir Osmangazi University Faculty of Medicine, Department of Hematology, Eskişehir, Turkey; 2 İstinye University Medical School, Department of Hematology, İstanbul, Turkey; 3 Ankara Yıldırım Beyazıt University Medical School, Department of Hematology, Ankara, Turkey; 4 Başkent University Faculty of Medicine, Department of Hematology, Ankara, Turkey

**Keywords:** Castleman disease, Diagnosis, Treatment

## Abstract

Castleman disease is a rare lymphoproliferative disease also known as angiofollicular lymph node hyperplasia. It is classified as hyaline vascular and plasmacytic variants histologically but characteristics of both types can coexist. Most unicentric cases of the disease are hyaline vascular while most multicentric cases are of the plasmacytic type. Although the pathogenesis is not completely understood, the role of interleukin (IL)-6 in unicentric disease and the roles of IL-6 and human herpes virus-8 in multicentric disease are well defined. Unicentric disease is typically localized and symptoms are minimal and treated locally. Multicentric disease is systemic and clinically characterized by generalized lymphadenopathy, splenomegaly, anemia, and systemic inflammatory symptoms. Systemic therapies are primarily given. Several malignant diseases including lymphomas, POEMS syndrome, follicular dendritic cell sarcomas, paraneoplastic pemphigus, Kaposi sarcoma, and amyloidosis can be associated with Castleman disease. In this paper, recent information about Castleman disease, which is a rare disease, is summarized.

## Abstract (tr)

Castleman hastalığı, anjiyofolliküler lenf nodu hiperplazisi olarak da bilinen nadir bir lenfoproliferatif hastalıktır. Histolojik olarak hiyalin vasküler ve plazmasitik varyant olarak sınıflandırılır ancak nadiren iki tipe ait özellikler bir arada bulunabilir. Unisentrik hastalığı olan olguların çoğu hiyalin vasküler, multisentrik hastalığı olan olguların çoğu ise plazma hücreli histolojik tipindedir. Patogenezi tam olarak anlaşılmamıştır fakat unisentrik hastalıkta interlökin (IL)-6’nın, multisentrik hastalıkta IL-6 ve human herpes virüs-8’in rolü iyi tanımlanmıştır. Unisentrik hastalık tipik olarak lokalizedir, semptomlar minimaldir ve tek başına lokal tedavi uygulanır. Multisentrik hastalık sistemik bir hastalıktır ve klinik olarak yaygın lenfadenopati, splenomegali, anemi ve sistemik inflamatuar semptomlarla karakterizedir. Başlıca sistemik tedaviler uygulanır. Lenfomalar, POEMS sendromu, folliküler dendritik hücreli sarkomlar, paraneoplastik pemphigus, Kaposi sarkomu, amiloidoz Castleman hastalığı ile ilişkili olabilir. Bu yazıda nadir bir hastalık olan Castleman hastalığı ile ilgili güncel bilgiler özetlenmiştir.

## Introduction

Castleman disease (CD), also known as angiofollicular lymph node hyperplasia and giant lymph node hyperplasia, was first reported by Benjamin Castleman in 1954. It is a rare disease diagnosed in 6600-7700 individuals each year in the United States [1,2].

CD is classified as unicentric CD (UCD), involving a single lymph node or a single region of nodes, and multicentric CD (MCD), involving multiple lymphatic regions [3]. UCD is more common [1] and has been reported to occur in younger individuals than MCD [4,5,6,7,8]. MCD can occur in any region of the body and has poorer prognosis.

**Figure 1:**
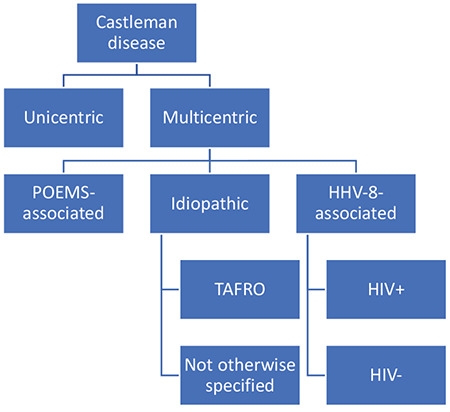
Classification of Castleman disease. POEMS: Polyneuropathy, organomegaly, endocrinopathy, monoclonal protein, and skin changes; HHV-8: human herpesvirus-8; TAFRO: thrombocytopenia, anasarca, fever, reticulin fibrosis, and organomegaly; HIV: human immunodeficiency virus.

MCD is further divided into three subgroups: human herpes virus-8 (HHV-8)-associated MCD; polyneuropathy, organomegaly, endocrinopathy, monoclonal gammopathy, and skin changes (POEMS)-associated MCD; and idiopathic MCD (iMCD) (Figure 1) [9].

## Diagnosis

Standard investigations for CD usually begin with lymph node biopsy followed by radiological investigation, preferably with positron emission tomography/computed tomography (PET/CT), complete blood count, serum chemistry, markers of inflammation, serum cytokine levels, viral serology for HHV-8 and human immunodeficiency virus (HIV), protein electrophoresis, immunofixation, and quantitative immunoglobulins [10,11].

The diagnostic criteria for iMCD and TAFRO syndrome, explained below, are summarized in Table 1 [12]. Diagnosis of HHV-8-associated MCD requires HHV-8 detection and plasmablastic histopathologic findings on lymph node biopsy [13]. POEMS-associated MCD is diagnosed if only one of the two mandatory major criteria of polyneuropathy and monoclonal plasma proliferative disorder is present with lymph node biopsy diagnostic of CD [14].

## Differential Diagnosis

Autoimmune diseases (immunoglobulin G4-related disease, rheumatoid arthritis, systemic lupus erythematosus, adult-onset Still disease), neoplastic disorders (lymphoma, desmoid tumors, retroperitoneal sarcoma, paragangliomas, sarcomas, hemangiopericytoma, bronchial adenoma, neurofibroma, chest wall tumors, schwannoma), and infectious disorders (HIV, Epstein-Barr virus [EBV], cytomegalovirus, tuberculosis, toxoplasmosis) must be considered in the differential diagnosis [3,14,15,16,17,18,19].

## Pathogenesis

Excessive cytokine production underlies the pathogenesis of CD. UCD and POEMS-associated MCD are caused by somatic mutations in monoclonal stromal and plasma cells [20]. In HHV-8-associated MCD, HHV-8 leads to a viral cytokine storm driven by interleukin-6 (IL-6) [11,12,21,22]. The exact mechanism of iMCD is unknown, but elevated IL-6 associated with autoimmune mechanisms, ectopic cytokine secretion by tumor cells, and/or viral signaling by a non-HHV-8 virus have been proposed [23].

## Pathology

The types of CD (hyaline vascular or hypervascular, plasmacytic, and mixed) are characterized by distinctive lymphoid architectural changes in all nodal compartments. The hyaline vascular variant is the most common type of UCD. MCD is predominantly of the plasmacytic variant with a few cases showing plasmablastic characteristics (Table 2) [24].

## Clinical and Laboratory Features

UCD may be clinically silent and laboratory findings are typically unremarkable. On the other hand, MCD presents with diffuse lymphadenopathy, systemic inflammation, and organ dysfunction [25]. Comorbid malignancies, lymphoma in iMCD, and Kaposi sarcoma in HHV-8-associated MCD have been described [26,27,28]. Patients with MCD may demonstrate anemia, leukocytosis, thrombocytopenia, thrombocytosis, elevated C-reactive protein, elevated IL-6, elevated erythrocyte sedimentation rate, elevated IgG, hypoalbuminemia, renal dysfunction, and elevated liver enzymes [1,26]. Clinical and laboratory features of CD are summarized in Table 3.

## Specific Presentations of Castleman Disease Paraneoplastic Pemphigus

The presence of mouth ulceration is highly suggestive of pemphigus and the severity of the disease correlates with lung involvement. It is more frequent in the context of UCD [11].

## POEMS Syndrome

POEMS syndrome refers to the presence of peripheral neuropathy, organomegaly, endocrinopathy, monoclonal gammopathy, and skin changes. Other frequent clinical findings are papilledema, pleural effusions, ascites, sclerotic bone lesions, and thrombocytosis [21].

## TAFRO Syndrome

TAFRO syndrome corresponds to a subtype of iMCD characterized by thrombocytopenia (T), anasarca (A), fever (F), reticulin fibrosis (R), and organomegaly (O) [11]. The outcome may be worse than in other cases of iMCD and no specific treatment has been identified [22]. Diagnostic criteria for TAFRO syndrome are summarized in Table 4.

## Hemophagocytic Lymphohistiocytosis

MCD and especially HHV-8-related MCD may be characterized by hemophagocytic lymphohistiocytosis at the initial presentation or upon relapse [25,26].

## Autoimmune Cytopenia

Autoimmune hemolytic anemia is a relatively frequent complication of MCD. Immune thrombocytopenia has been reported in 5% to 20% of MCD cases [25,27,28].

## Peripheral Neuropathy

Demyelinating peripheral neuropathy is frequently observed with CD. There is no clear association between the severity of the peripheral neuropathy and the subtype of CD [29].

## Renal Involvement

Renal involvement is frequently observed in MCD, mainly in the plasmacytic and mixed subtypes, being reported in up to 25% of MCD cases. Glomerular lesions, AA amyloidosis, and interstitial nephritis are the most common renal pathology findings [30].

## Treatment

Surgical resection provides radical treat­ment for the majority of patients with UCD. Radiotherapy is an important alternative when surgical resection is contraindi­cated or technically difficult. Other treatment options are embolization, rituximab, or siltuximab/tocilizumab in the event of inflammation [23].

Treatment of MCD still remains complex because MCD is a rare clinical entity and there is a lack of randomized controlled trials. Multiple therapeutic approaches have been used, including conventional cytotoxic chemotherapy (single-agent or combined), antiviral treatment, glucocorticoids, thalido­mide, interferon-alpha, and molecular targeted therapies. De­termination of HHV-8 status is also important [10]. Therapeutic approaches for MCD are listed in Table 5.

The use of prednisone or other glucocorticosteroids will frequently ameliorate symptoms, partially improve lymphadenopathy, and correct laboratory abnormalities. However, the impact is generally temporary. Lasting remissions are rare and the disease may require the long-term use of corticosteroids, increasing the risk of bacterial infections [31,32]. The use of corticosteroids alone may be better reserved as a temporary intervention in acute situations where more definitive therapy has not yet been decided or will be delayed [24].

Currently, chemotherapy is the first option for most symptomatic patients. However, data are insufficient to favor one treatment for all patients. Oral chlorambucil and cyclophosphamide have been effective and are generally well tolerated [23,31,33]. Vinblastine [10] and oral etoposide [13] may also have activity. Therapy with a single alkylating agent may be most appropriate for fragile patients or cases in which a prompt response is not required.

**Figure 2:**
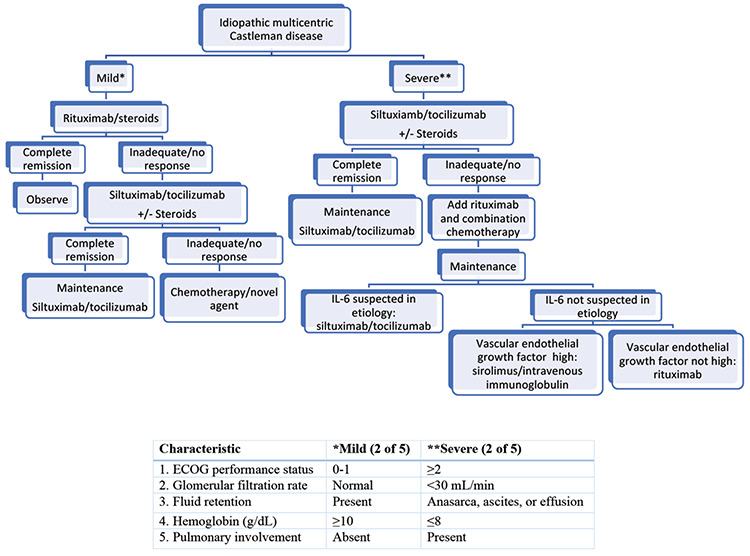
Treatment algorithm for idiopathic multicentric Castleman disease. ECOG: Eastern Cooperative Oncology Group.

Combination chemotherapy regimens such as cyclophosphamide, vincristine, and prednisone or cyclophosphamide, doxorubicin, vincristine, and prednisone have significant activity [10,14,31]. When combination chemotherapy is used, patients need to be closely monitored because of the increased risk of infection. Patients with HIV-associated CD may be at especially high risk for complications with standard combination chemotherapy [34]. A treatment algorithm is shown in Figure 2 [34,35].

## Conclusion

CD is a rare lymphoproliferative disease that can mimic many malignant and nonmalignant conditions. Lymph node biopsy is essential to establish a definitive diagnosis and greater awareness of the disease among clinicians would facilitate early diagnosis.

## Figures and Tables

**Table 1:**
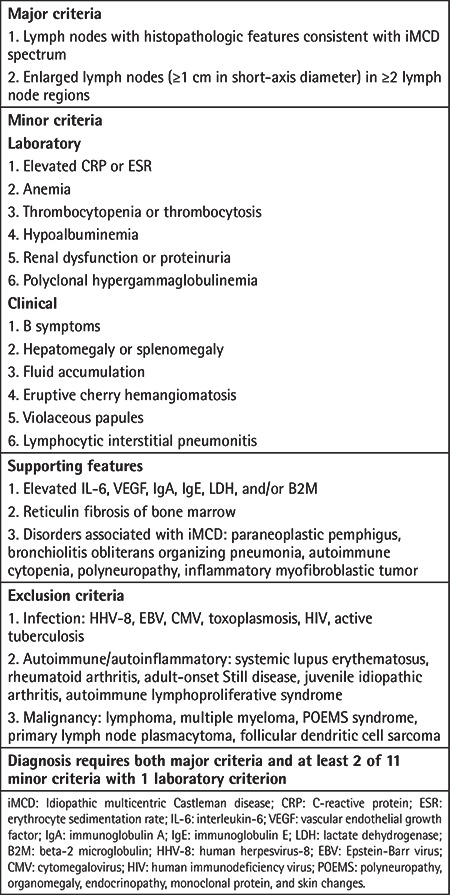
Diagnostic criteria for idiopathic multicentric Castleman disease.

**Table 2:**
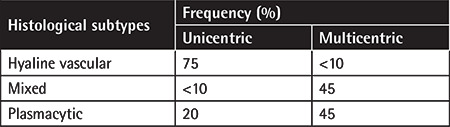
Histopathology of Castleman disease.

**Table 3:**
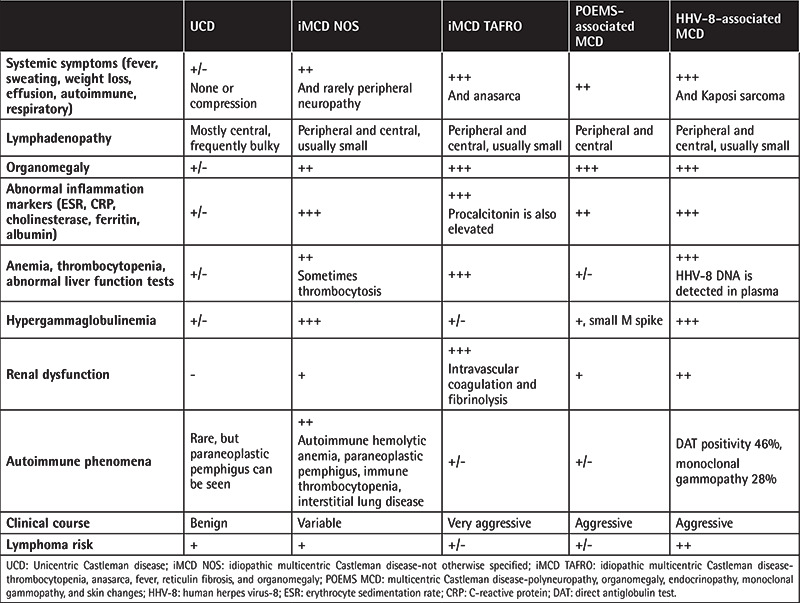
Clinical and laboratory features of Castleman disease.

**Table 4:**
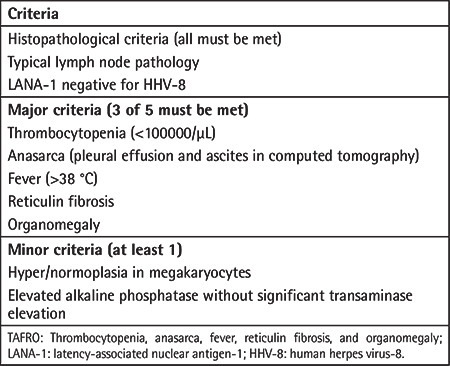
Diagnostic criteria for TAFRO syndrome.

**Table 5:**
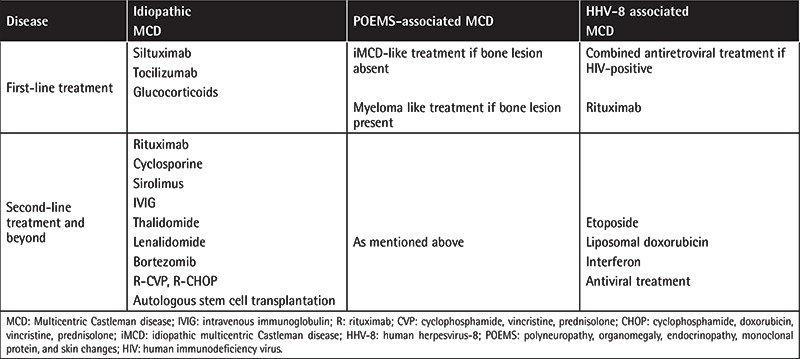
Therapeutic approaches for multicentric Castleman disease.
